# Transcriptome and Metabolome Analyses Reveal That Nitrate Strongly Promotes Nitrogen and Carbon Metabolism in Soybean Roots, but Tends to Repress It in Nodules

**DOI:** 10.3390/plants7020032

**Published:** 2018-04-12

**Authors:** Shinji Ishikawa, Yuki Ono, Norikuni Ohtake, Kuni Sueyoshi, Sayuri Tanabata, Takuji Ohyama

**Affiliations:** 1 Graduate School of Science and Technology, Niigata University, Niigata 950-2181, Japan; s.ishikawa@asahi-kg.co.jp (S.I.); f15d021h@outlook.jp (Y.O.); ohtake@agr.niigata-u.ac.jp (N.O.); sueyoshi@agr.niigata-u.ac.jp (K.S.); 2 Faculty of Agriculture, Ibaraki University, Ibaraki 300-0332, Japan; sayuri.tanabata.i@vc.ibaraki.ac.jp; 3 Faculty of Applied Biosciences, Tokyo University of Agriculture, Tokyo 156-8502, Japan

**Keywords:** metabolome, microarray, nitrate, nitrogen fixation, nodule, root, soybean, transcriptome

## Abstract

Leguminous plants form root nodules with rhizobia that fix atmospheric dinitrogen (N_2_) for the nitrogen (N) nutrient. Combined nitrogen sources, particular nitrate, severely repress nodule growth and nitrogen fixation activity in soybeans (*Glycine max* [L.] Merr.). A microarray-based transcriptome analysis and the metabolome analysis were carried out for the roots and nodules of hydroponically grown soybean plants treated with 5 mM of nitrate for 24 h and compared with control without nitrate. Gene expression ratios of nitrate vs. the control were highly enhanced for those probesets related to nitrate transport and assimilation and carbon metabolism in the roots, but much less so in the nodules, except for the nitrate transport and asparagine synthetase. From the metabolome analysis, the concentration ratios of metabolites for the nitrate treatment vs. the control indicated that most of the amino acids, phosphorous-compounds and organic acids in roots were increased about twofold in the roots, whereas in the nodules most of the concentrations of the amino acids, P-compounds and organic acids were decreased while asparagine increased exceptionally. These results may support the hypothesis that nitrate primarily promotes nitrogen and carbon metabolism in the roots, but mainly represses this metabolism in the nodules.

## 1. Introduction

Soybean is a globally important legume crop that provides proteins and lipids to humans and livestock. The annual production of soybean was estimated to be 335 M t in 2016 (FAOSTAT); it is the fourth most abundantly produced grain crop after maize, paddy rice, and wheat. Because soybean seeds contain a high concentration of protein, approximately 35–40%, they require relatively larger amounts of nitrogen than do cereal grains to obtain the same seed yield [1]. Soybean plants can form root nodules and utilize atmospheric N_2_ in association with rhizobia, which are nitrogen-fixing soil bacteria. Harper [2] previously reported on the importance of both N_2_-fixation by root nodules and inorganic nitrogen absorption by roots to obtain the optimum yield of soybeans. However, it is well known that nodulation and the nitrogen fixation activity of root nodules are suppressed when the nodulated roots are exposed to a high concentration of combined nitrogen—especially nitrate, a major form of inorganic nitrogen in the upland soil, which strongly inhibits nodulation and N_2_ fixation in soybean plants [3,4,5]. Nitrate inhibition is known to exert pleiotropic effects, including reductions in nodule numbers, nodule masses, and N_2_ fixation activity, as well as accelerating nodule senescence or disintegration [4,6,7,8,9,10,11]. The effects of nitrate inhibition on nodule growth and nitrogen fixation in soybeans are complex, however. The nitrate supply to nodulated roots can lead to negative or positive effects depending on the nitrate concentration, its placement in the soil, the growth medium, and the application period [12,13]. Previous studies reported that nitrate inhibition was primarily host plant-dependent and was independent of nitrate metabolism in the rhizobia [3,14]. Nevertheless, the mechanism underlying nitrate inhibition remains controversial. There are many hypotheses proposed for the reason why nitrate inhibits nodule growth and N_2_ fixation activity, such as, carbohydrate deprivation in nodules [9,15,16], feedback inhibition by the products of nitrate metabolism such as glutamine [17] or asparagine [18], and interrupt O_2_ diffusion into the nodules, which restricts the respiration and nitrogen fixation activity of bacteroids, the symbiotic form of rhizobia in the nodules [15,19]. More recently, the possible involvement of nitric oxide has also been reported in the inhibition of nitrogenase activity by nitrate in *Lotus* nodules [20].

We investigated the direct effects of nitrate on the nodule growth of soybean plants cultivated with hydroponics, in which the diameters of the horizontal axes of individual nodules were measured by a slide caliper [21] or the nodule area, fitted to an ellipse oval, was determined through photographs periodically taken by a digital camera [10]. Nodule growth and N_2_ fixation activity were quickly decreased by adding 5 mM of nitrate to a culture solution in a few days, whereas the inhibition of nodule growth and N_2_ fixation activity by nitrate was reversibly recovered by changing the 5 mM nitrate to 0 mM nitrate in the culture solution. After a 2-week treatment with 5 mM of nitrate, both nodule growth and nitrogen fixation activity (acetylene reduction activity) had completely recovered by a 1-week treatment with 0 mM nitrate [21]. These findings indicated that early senescence or irreversible disintegration did not occur in soybean nodules treated for at least for 2 weeks with 5 mM nitrate. These nodules maintained their functions for growth and nitrogen fixation even though nodule growth was temporally stopped entirely in the presence of nitrate. Further experiments, in which soybean shoots were exposed to ^11^C or ^14^C-labeled CO_2_, were performed to investigate the effects of nitrate supply on the translocation and distribution of newly assimilated photosynthates to the nodules and roots of soybean [10]. The addition of 5 mM of nitrate to the culture solution stimulated the translocation of labeled C to the roots from 5.2% to 9.1% of total photoassimilate and decreased the partitioning of C to the nodules from 9.1% to 4.3% [10]. These findings indicate that a decreased supply of photoassimilates to nodules might be involved in the quick but reversible nitrate inhibition of nodule growth and its nitrogen fixation activity.

Recently, the effects of nitrate on soybean nodule growth were monitored at 1-h intervals, and the volume of nodules was measured with newly developed computer software “Nodame” [22,23]. Nodule growth began to be suppressed at about 7-h after the addition of 5 mM of nitrate compared with control under light conditions. In addition, the nodule growth rate under dark conditions was decreased more severely, reaching only almost half that occurring under lit conditions. The nodule growth was additively suppressed by the addition of the 5 mM nitrate to the medium in the dark. Similar repressive effects were observed on the growth rate of the primary root with a 5-mM nitrate under light/dark conditions. Conversely, the growth of soybean lateral roots was promoted by the addition of 5 mM of nitrate [22].

Ruffel et al. [24] reported the transcriptome analysis of split nodulated roots and shoot comparing various N sources in *Medicago trancatula*. The effects of nitrate on gene expression in the nodules of *Medicago truncatula* were recently published in a study that used RNA-sequencing transcriptome analysis [25]. After 4- and 28-h nitrate treatments, the expression of 127 genes for the nodule-specific cysteine-rich peptides and leghemoglobin, as well as the genes related to iron allocation and mitochondrial ATP synthesis, were all downregulated, while the expression of genes related to nitrate transport and nitrate reduction were upregulated. In that research work, however, gene expression in the roots was not analyzed, and the mechanism of nitrogen inhibition may not be the same as for *Glycine max*, as it could differ among legume species and determinate (soybean) and indeterminate (*Medicago trancatula*) types of nodules. The present study is the first report of transcriptome and metabolome analyses that compare the roots and nodules in relation to the nitrate inhibition of nitrogen-fixing soybean nodules.

The number of legume nodules is systemically regulated by the previous rhizobial infections in order to avoid excessive nodulation, and this phenomenon is called the autoregulation of nodulation (AON) [26,27,28]. Several lines of hypernodulation or supernodulation mutants that lacked AON have been isolated and exhibited profuse nodulation compared with parent lines [29,30,31,32]. A reciprocal shoot and root grafting experiment, by using a hypernodulation mutant line and the wild-type, revealed that AON was controlled by the shoot genotype, and not the roots [31]. It is interesting that all hypernodulation mutant lines so far showed partial tolerance to nitrate for their nodule formation, thus a common mechanism may be shared between the nitrate inhibition of nodulation and AON [29,30,31,32]. Genes responsible for AON, such as *LjHAR1* have been identified in the model legume *Lotus japonicus* and *GmNARK* in the soybean, have been identified (reviewed by Soyano et al. [28]). Small peptides, named CLAVATA3/EMBRYO SURROUNDING REGION-RELATED (CLE) peptides, may be candidates of the infection signal from the roots to the shoot, based on similarities between the CLAVATA1 and CLE peptide signaling in *Arabidopsis thaliana*, which controls shoot meristem development [27,28] in the form of arabinosylated glycopeptides [33]. Collectively, these findings suggest that an inhibitor is synthesized in the shoot when NARK proteins receive the CLE peptides; and the shoot-derived unknown inhibitor is then transported from the shoot to the roots, where it prevents the differentiation and development of the nodule primordia. However, there is no direct evidence to link the nitrate inhibition and AON.

## 2. Results

### 2.1. Number and Function of Upregulated and Downregulated Genes in Roots and Nodules

**Figure 1:**
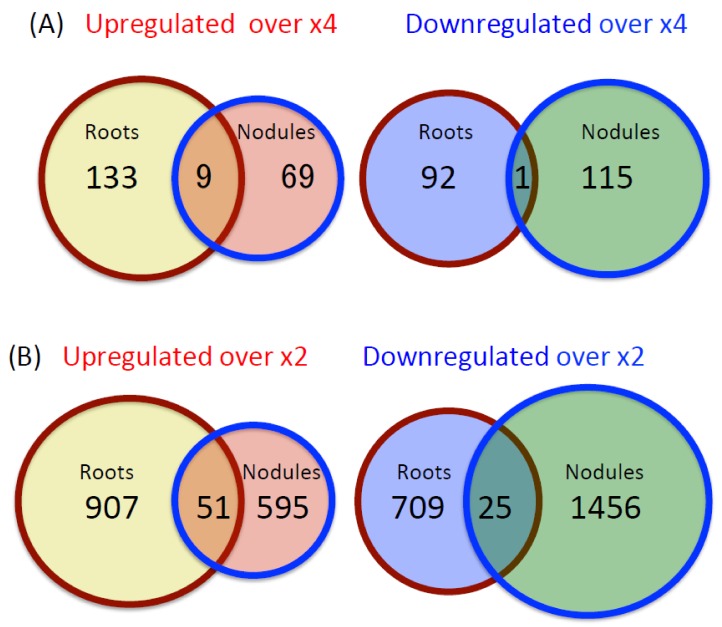
Numbers of probesets in soybean roots and nodules upregulated or downregulated genes by more than ×4 or ×2 following the addition of 5 mM of nitrate to the medium for 24 h, at the level of *p* < 0.05 (Student’s *t*-test). Data were obtained from three independent biological replications. (**A**) Numbers of probesets in the roots and nodules upregulated or downregulated genes by more than fourfold. (**B**) Numbers of probesets in the roots and nodules upregulated or downregulated genes by more than twofold.

The number of upregulated or downregulated genes in the roots and nodules, at 24 h after the addition of 5 mM nitrate as compared with control plants, is shown in Figure 1A (more than fourfold, *p* < 0.05) and Figure 1B (more than twofold, *p* < 0.05). We found that 142 and 78 probesets that were highly upregulated by more than fourfold in the roots and nodules, respectively, of which nine probesets were common to both roots and nodules. By contrast, the expression of 93 and 116 probesets was highly downregulated by more than fourfold in the roots and nodules, respectively, with only one probeset in common to both plant organs. The expression of 958 and 646 probesets were upregulated by more than twofold in the roots and nodules, respectively, and 51 probesets were common in the roots and nodules. On the other hand, the expression of 734 and 1481 probesets was downregulated by more than twofold in the roots and nodules, respectively, with only 25 probesets in common to both plant organs. The number of upregulated probesets were higher in the roots than that in the nodules using both the fourfold and twofold criterions. Conversely, the number of downregulated probesets was higher in the nodules than in the roots.

**Figure 2:**
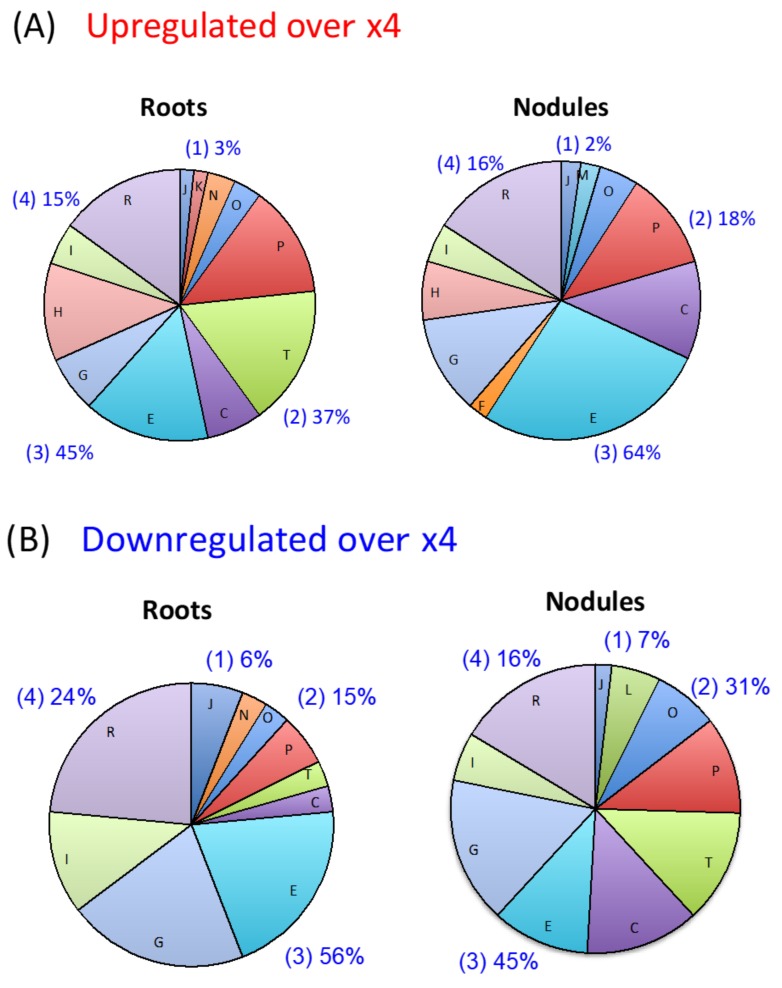
Functional distribution of genes differentially expressed in soybean roots and nodules by the 5-mM nitrate treatment. (**A**) Upregulated genes > fourfold in the roots and nodules of soybean plants after 24 h of the 5 mM of the nitrate treatment compared with control. (**B**) Downergulated genes > fourfold in the roots and nodules of soybean plants after 24 h of the 5 mM of the nitrate treatment compared with control. Functional categories: (1) Information storage and processing, J: translation, ribosomal structure and biogenesis, K: transcription, L: DNA replication, recombination, and repair. (2) Cellular processes, D: cell division and chromosome partitioning, M: cell envelope biogenesis, outer membrane, N: cell motility and secretion, O: post-translational modification, protein turnover, chaperones, P: inorganic ion transport and metabolism, T: signal transduction mechanisms. (3) Metabolism, C: energy production and conversion, E: amino acid transport and metabolism, F: nucleotide transport and metabolism, G: carbohydrate transport and metabolism, H: coenzyme transport and metabolism, I: lipid metabolism. (4) Poorly characterized proteins, R: general function prediction only.

The functional annotation of soybean genes on this array was obtained from 22,674 scaffolds [34], with the percentages of genes classified into the information storage and processing, cellular processing and signaling, metabolism, and poorly characterized being 19%, 32%, 33% and 16%, respectively. For the functional categories of metabolism, energy production and conversion, carbon transport and metabolism, and amino acid transport and metabolism were 7%, 7%, and 5%. The percentage of probesets upregulated more than fourfold in the roots and nodules by the nitrate treatment were classified as information storage and processing (3% in roots and 2% in nodules), cellular processes (37% in roots and 18% in nodules), metabolism (45% in roots and 64% in nodules), and poorly characterized (15% in roots and 16% in nodules) (Figure 2A). The percentage of downregulated probesets more than fourfold in the roots and nodules were related to information storage and processing (6% in roots and 7% in nodules), cellular processes (15% in roots and 31% in nodules), metabolism (56% in roots and 45% in nodules), and poorly characterized (24% in roots and 16% in nodules) (Figure 2B). The regulation was most pronounced for metabolism followed by cellular processes, both in the roots and nodules irrespective of the upregulated or downregulated genes more than fourfold. Under “metabolism” amino acid metabolism (E), carbohydrate transport and metabolism (G), and energy production and conversion (C), were relatively dominant. Under “cellular processes” signal transduction (T), and inorganic ion transport and metabolism (P) were relatively dominant.

The lists of upregulated (Table S1 for roots, Table S2 for nodules) or downregulated (Table S3 for roots, Table S4 for nodules) genes over twofold are given. The high ratios of upregulated genes in the roots after the nitrate treatment (Table S1) were annotated as *Lj* (*Lotus japonicus*) ferredoxin-nitrite reductase (NiR) (1315, 535, 216 fold; for each probeset); *Gm* (*Glycine max*) nitrate reductase (NR) (79, 63, 52 fold); *Gm* glutamine synthetase (GS) (24.1, 23.8, 16.9, 6.9 fold); the *Gm* high affinity nitrate transporter (NRT2) (23.1 fold); *Gm* phosphoenolpyruvate carboxylase (PEPC4) (18.9, 6.5 fold); glucose-6-phosphate 1-dehydrogenase (11.7, 10.7, 10.6 fold); *Pv* (*Phaseolus vulgaris*) NADH glutamate synthase (11.0-fold); *Gm* asparagine synthetase 1 (AS1) (9.8-, 9.7-, 8.8-, 6.4-fold); and *Gm* asparagine synthetase 2 (AS2) (8.3-, 5.5-fold) genes. In general, most of these genes were related to nitrate transport, nitrogen assimilation, and the carbon metabolism associated with nitrogen metabolism. The high ratios of gene expression of the upregulated genes in the nodules after the nitrate treatment (Table S2) were annotated as aspartylprotease genes (23.0-, 6.1-fold); *Gm* alcohol dehydrogenase 1 (8.6-fold); *Gm* tyrosine aminotransferase (8.3-fold); the *Gm* genes induced by salicylic acid (8.0-, 5.5-, 5.1-fold); *Gm* resistant protein KR3 (7.5-fold); the *Gm* high affinity nitrate transporter (NRT2) (6.8-fold); *Gm* beta-amylase (BMY2) (5.6-fold); *Gm* proline dehydrogenase (5.4-fold); the *Gm* auxin-regulated protein (Aux22) (5.0-fold); *Gm* proline dehydrogenase (PDH) (4.7 fold); *Gm* asparagine synthetase (AS1) (4.7-, 4.6-, 4.3-fold); the *Gm* chlorophyll ab-binding protein (CAB3) (4.0-fold); and the *Gm* riburose-1,5-bisphosphate carboxylase small subunit (RBCS3) (4.0-fold) genes; most of which were not related to nitrate transport or nitrogen assimilation. Commonly shared probesets, the expression of which was affected more than fourfold in both the roots and nodules, were the *Gm* putative nitrate transporter (NRT2), H^+^ oligopeptide symporter, Cl^−^ channel, and *Gm* asparagine synthetase 1 (AS1).

The high ratios of downregulated gene expression in the roots after nitrate treatment (Table S3) were annotated as *Gm* β-amylin synthase (AMS1) (13.3-fold); *Gm* glutathione S-transferase (GST5) (11.6-fold); *Gm* chitinase III-A (5.1-fold); and a *Gm* gene induced by salicylic acid (4.4-fold). The high ratios of downregulated gene expression in the nodules after nitrate treatment (Table S4) were annotated as *Gm* asparaginase (14-, 12-fold); and a serine threonine salicylic acid (10-fold). Only one gene, the *Gm* gene induced by salicylic acid, was commonly reduced in both roots and nodules.

### 2.2. Upregulation and Downregulation of Root-Specific and Nodule-Specific Genes by the Nitrate Treatment

Root- and nodule-specific probesets were selected by the ratios of gene expressions in roots vs. nodules using the cutoff criterion of a twofold change. Of 42,748 probesets analyzed, 10,842 and 9236 probesets were respectively root-specific and nodule-specific genes in our experiment. The expression of 53 root-specific genes in the nitrate-treated roots (Table S5) was upregulated more than twofold by the 5-mM nitrate treatment, whereas almost 10 times as many (491) root-specific genes (Table S6) were downregulated. Concerning the nodule-specific genes, the expression of 116 probesets were upregulated while 392 were downregulated. The root-specific genes upregulated by nitrate contain mth2, aquaporin, glutathione S-transferase, NRT2, a salicylic acid-induced gene, amino acid transporter, Myb family transcription factor and so on (refer to Table S5). The root-specific genes downregulated by nitrate included asparaginase, ammonia permease, cytochrome P450 CYP2 subfamily, aspartyl protease and so on. (refer to Table S6). The nodule-specific genes promoted by nitrate included β-amylase (bmy2), asparagine synthetase 1 (AS1), lipoxygenase-1, trehalose-6-phosphate synthase component TPS1, Zn-finger protein, and so on. (refer to Table S7). The nodule-specific genes repressed by nitrate include fructose-1,6-bisphosphatase, tyrosine aminotransferase, early nodulin GmN 70, dehydration-responsive element-binding protein 2C (DREB 2C protein), amino acid transporters, aquaporin and so on (refer to Table S8).

### 2.3. Upregulation and Downregulation of Leghemoglobin and Nodulin Genes by the Nitrate Treatment

The effect of nitrate on leghemoglobin (Table S9) and nodulin (Table S10) genes in soybean roots and nodules were evaluated. Nodulins are defined as plant-encoded proteins that are specifically expressed during nodule development [35]. All six leghemoglobin probesets, which encodes four leghemoglobin components—LbA, LbC1, LbC2 and LbC3—were highly expressed in the nodules and much less so in the roots, and the ratios of nodule/roots in the control plants were ranged from 25–176-fold (Table S9). However, the expression of leghemoglobin genes was not affected by the nitrate treatment, for which the ratio of nitrate vs. control was about 1.1-fold and statistically not significant. The same ratio in the roots tended to increase to about 2-fold, though also not significant.

Eighty-eight nodulin genes were found (Table S10). Of these, thirteen probesets were enhanced while six were repressed by the nitrate treatment in the roots, and likewise, seven probesets were enhanced while four probesets were repressed in the nodules over twofold. Early nodulin 40 and nodulin 21 were similarly enhanced in the roots and nodules. Early nodulin gene expressions of GmN70, GmN93, and GmN315 were significantly decreased in the nodules treated with nitrate. Although statistically not significant, nodulin 35, which encodes nodule-specific uricase II, was highly enhanced (by >10-fold) in the roots.

### 2.4. Upregulation and Downergulation of Phytohormone-Related Genes by the Nitrate Treatment

Supplying nitrate to nodulated soybean plants affects the growth of their roots and nodules [36]. Hence, the nitrate treatment may have influenced hormone synthesis or signal transductions. However, the effect of the nitrate treatment on the expression of auxin, cytokinin, gibberellin, abscisic acid, and ethylene-related probesets was not remarkable after 24 h of 5 mM nitrate treatment, since their ratios were close to a value of 1-fold. Figure S1 shows the effect upon auxin-regulated probesets. Most probesets were not drastically affected by the nitrate treatment, neither in the roots nor nodules, except for three homologs of AuxIAA genes that had a more than a twofold change.

### 2.5. Metabolome Analysis of Roots and Nodules Comparing the Nitrate Treatment and Control

A total of 315 compounds (168 compounds by cation analysis, and 147 compounds by anion analysis) were detected in the metabolome analysis (Table S11). The concentration of 91 compounds (47 cationic compounds and 44 anionic compounds) were quantified and shown in Table S12. The concentrations of Glu (295 nmol gFW^−1^ in roots and 3320 nmol gFW^−1^ in nodules), Asp (224 nmol gFW^−1^ in roots and 2330 nmol gFW^−1^ in nodules), Asn (423 nmol gFW^−1^ in roots and 449 nmol gFW^−1^ in nodules), Gln (137 nmol gFW^−1^ in roots and 576 nmol gFW^−1^ in nodules), Ser (201 nmol gFW^−1^ in roots and 1685 nmol gFW^−1^ in nodules), were relatively high among free amino acids in control treatment. Among organic acids the concentrations of malic acid was highest both in roots (2390 nmol gFW^−1^) and nodules (5840 nmol gFW^−1^), followed by succinic acid, citric acid, glucronic acids both in the roots and nodules. Among sugar phosphates, the concentration of glucose-6-phosphate (237 nmol gFW^−1^ in roots and 771 nmol gFW^−1^ in nodules), was highest both in roots and nodules. The concentrations of ATP (3.2 nmol gFW^−1^ in roots and 13 nmol gFW^−1^ in nodules), ADP (4.4 nmol gFW^−1^ in roots and 31 nmol gFW^−1^ in nodules) were relatively lower than those of amino acids and organic acids.

When the concentration of each compound was compared with nodules and roots in control treatment without nitrate (Table S13), the metabolite concentration are generally higher in the nodules than those in the roots: the ratios (nodules/roots) of five compounds were over 100-fold, 45 compounds were between 10-fold and 100-fold, 87 compounds were between 2-fold and 10-fold, 22 compounds were between 1-fold and 2-fold significantly. The ratios significantly lower than 1-fold were only 15. The high ratios of compounds in nodules/roots in control treatment were *N*-acetylputrescine (239-fold), adenine (219-fold), *N*-acetyl ornithine (138-fold), *N*-acetylglutamic acid (127-fold), *N*-methylputrescine (103-fold), 4-guanidinobutyric acid (73-fold), citramalic acid (53-fold), arginosuccinic acid (43-fold), histidinol (42-fold), 2-deoxyglucose 6-phosphate (37-fold), 3′-AMP (37-fold), cytidine (35-fold), sedheptulose 7-phosphate (32-fold), uridine (29-fold), *N*6-acetyllysine (26-fold), 2-isopropylmalic acid (28-fold), hydroxylproline (28-fold), allantoic acid (26-fold), citruline (23-fold), nicotinic acid (23-fold), spermidine (22-fold), hexanoic acid (22-fold), *N*-acetylserine (22-fold), glyceric acid 19-fold)*, N*^6^-acetylspermidine (17-old), trehalose 6-phosphate (16-fold), fumaric acid (15-fold), 6-phosphogluconic acid (14-fold), UDP (13-fold), 5-amino-4-oxovaleric acid (13-fold), hypoxanthicne (13-fold), glutamic acid (11-fold), NAD+ (11-fold), ascorbate 2-glucoside (11-fold), homoserinelactone (11-fold), aspartic acid (10-fold), were higher than 10-fold. The ratios lower than 1-fold were cis-aconiteic acid (0.28-fold), pyrophosphate (0.29-fold), ethyl glutcuronide (0.32-fold), AMP (0.39-fold), UDP-glucose and UDP-glucose (0.52-fold), UMP (0.56-fold), 3-amino-2-piperidone (0.55-fold), cAMP (0.56-fold), glycerol (0.63-fold), urocanic acid (0.63-fold).

In the roots (Table S14), seventeen compounds had a greater than twofold increase in response to nitrate (*15: * indicates the number of compounds at *p* < 0.05); with 1.5-fold to 2.0-fold ratios, it was 16 (*12); 1.1- to 1.5-fold was 61 (*17); 0.9- to 1.1-fold ratios, it was 68 (*0); 0.5- to 0.9-fold ratios, it was 43 (*7); and ratios lower than 0.5-fold, it was 6 (*3). The respective corresponding ratios for the number of compounds in the nodules (Table S15) were as follows: 6 (*3), 15 (*7), 79 (*14), 120 (0), 78 (*19), and 3 (*1). The number of compounds with a ratio more than twofold therefore much greater in the roots than in the nodules.

Table 1, Table 2 and Table 3 shows a comparison of the ratios of nitrate treatment over control of the selected compounds, as grouped by nitrogen compounds (Table 1), phosphorous compounds (Table 2), and organic acids (Table 3) in the roots and nodules. In the roots, the nitrogen compounds related to initial nitrate metabolism, namely Gln (2.4-fold) and Glu (1.8-fold) were high. The ratios of other amino acids, namely Asn (2.1-fold), Ala (2.0-fold) and Asp (1.8-fold), were also high. The ratio of allantoin was just 1.0-fold, whereas that of allantoic acid reached 3.1-fold. In the nodules, the nitrogen compounds related to initial nitrate metabolism were Gln (1.6-fold) and Glu (0.9-fold). The ratios of Asn (3.3-fold) and *N*-acetylglucosamine (3.6-fold) were high, whereas those of Ala (0.9-fold) and Asp (1.4-fold) were not so high. In the nodules, the ratios of allantoin (1.9-fold) and allantoic acid (1.4-fold) were higher than 1.0-fold.

Ratios of the phosphorous compounds in the roots were generally higher than 2-fold, especially for ATP (3.9-fold), which was the highest, followed by ADP (2.9-fold) and NAD (2.2-fold). By contrast, in the nodules, the concentration ratios of most compounds, such as ATP (0.7-fold), ADP (0.9-fold), and NAD (0.9-fold), were lower than 1.0-fold, with the sole exception of AMP (1.3-fold). These results indicated that energy metabolism in the roots was enhanced by the 5-mM nitrate treatment, whereas it might be repressed in the nodules. The ratios of organic acids were also increased by nitrate in the roots, but not in the nodules.

### 2.6. Nitrate Effect of Gene Expression and Metabolite Concentration on Nitrogen and Carbon Metabolism in Roots and Nodules

**Figure 3:**
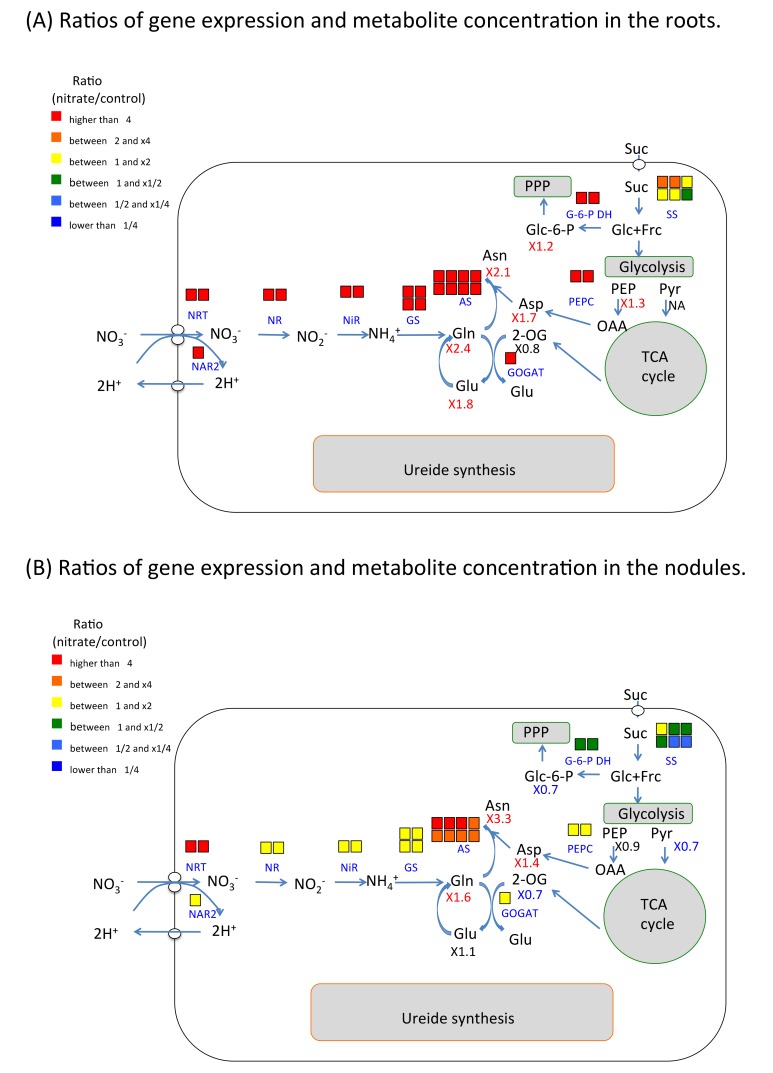
Ratios of gene expression and metabolites related to primary nitrogen assimilation and related carbon metabolism in soybean roots and nodules after 24 h of the 5-mM nitrate treatment. (**A**) Ratios of gene expression and metabolite concentration in the roots. (**B**) Ratios of gene expression and metabolite concentration in the nodules.

The ratios of gene expressions and metabolite concentrations with nitrate-treated/control related to nitrogen metabolism in the roots (Figure 3A) and nodules (Figure 3B) are shown in the metabolic map. The gene expression ratio of each probeset is indicated by colored square above the abbreviation of enzyme name. The concentration ratio of metabolite is shown under the metabolite abbreviation. In the roots, the expression of the genes especially those for nitrate transport and assimilation enzymes were markedly enhanced at 24 h after the 5-mM nitrate treatment. The corresponding root ratios of (Figure 3A) were as follows: NRT (48-, 23-fold) and NAR2 (13-fold) for nitrate transport through the cell membrane; NR (79-, 63-fold) and NiR (1315-, 535-fold) for nitrate-reducing enzymes; GS (24-fold to 7-fold) and GOGAT (11-fold) for ammonium-assimilating enzymes; and AS (9.8-fold to 5.3-fold) for the synthesis of asparagine. Likewise, the ratios of gene expression in the nodules (Figure 3B) were as follows; NRT (6.8-, 6.2-fold), NAR2 (1.8-fold), NR (1.4-, 1.3-fold), NiR (1.3-, 1.2-fold), GS (1.5-fold to 1.0-fold), GOGAT (1.5-fold), and AS (4.7-fold to 3.0-fold). Although nitrogen-assimilating genes were enhanced in the roots and nodules, their ratios were markedly higher in the former organ. The ratios of the nitrate-reducing enzymes, NR and NiR, as well as the ammonium-assimilating enzymes, GS and GOGAT, were affected by less than twofold in the nodules. In addition, the ratios of G-6-P DH (11.7-, 10.6-fold), PEPC (18.9-, 6.5-fold), and sucrose synthase (SS) (2.3-fold to 0.6-fold) in the roots were also higher than those in the nodules; (G-6-P DH (0.9-, 0.9-fold), PEPC (1.3-, 1.3-fold), and SS (1.2-fold to 0.8-fold)). The concentration ratios in the roots were significantly higher for Gln, Asn, Glu, Asp, PEP and Glc-6-P in the nitrate-treated roots. Those ratios were also high in nitrate-treated nodules in Asn, Gln and Asp, but lower in Pyr, 2-OG and Glc-6-P. These results indicated that the treatment with 5 mM of nitrate markedly enhanced the nitrate transport and nitrogen-assimilating enzymes in the roots. This change also occurred in the nodules but the expression ratios in this organ were relatively lower than seen in the roots.

The model shows the metabolic map of nitrogen and related carbon metabolism in the root or nodule cells. The color of the squares above each enzyme name indicates the fold strength of the ratio for the corresponding probesets. The number under the compound abbreviation indicate the concentration ratio of compounds. The red shows significant increase, blue shows significant decrease, and the black indicates notsignificant at *p* < 0.05.

**Figure 4:**
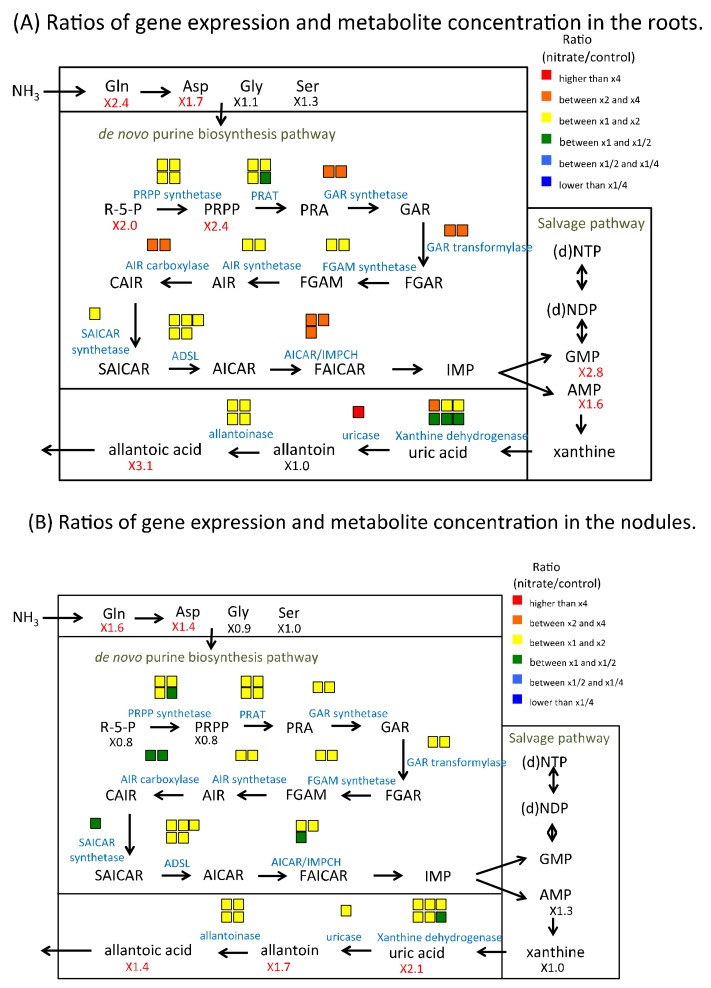
Ratios of gene expression related to ureide synthesis in soybean roots and nodules after 24 h of the 5-mM nitrate treatment. (**A**) Ratios of gene expression and metabolite concentrations in the roots. (**B**) Ratios of gene expression and metabolite concentrations in the nodules. The model shows the metabolic map of ureide synthesis in the root or nodule cells. The color of squares above enzyme name and the number under the compound abbreviation indicate the same as Figure 3.

Gene expression ratios for the synthesis of ureides, allantoin, and allantoic acid, in the roots were slightly enhanced (Figure 4A) from 1-fold to 2-fold, except for uricase (10.5-fold). These ratios were notably lower than those for the primary nitrogen-assimilating enzymes, as Figure 3A shows. The concentration ratio of allantoin was just 1.0-fold, but the ratio of allantoic acid was significantly higher (3.1-fold) in nitrate-treated roots over control. The ratios of gene expression related to purine biosynthesis and degradation in the nodules were nearly 1-fold (Figure 4B). The concentration ratios of uric acid (2.1-fold), allantoin (1.7-fold) and allantoic acids (1.4-fold) were significantly higher in nitrate-treated nodules over control nodules. Together, these results indicated that the 5-mM nitrate treatment for 24 h did not markedly affect the expression of genes for enzymes related to the synthesis of ureides in the roots and nodules.

**Figure 5:**
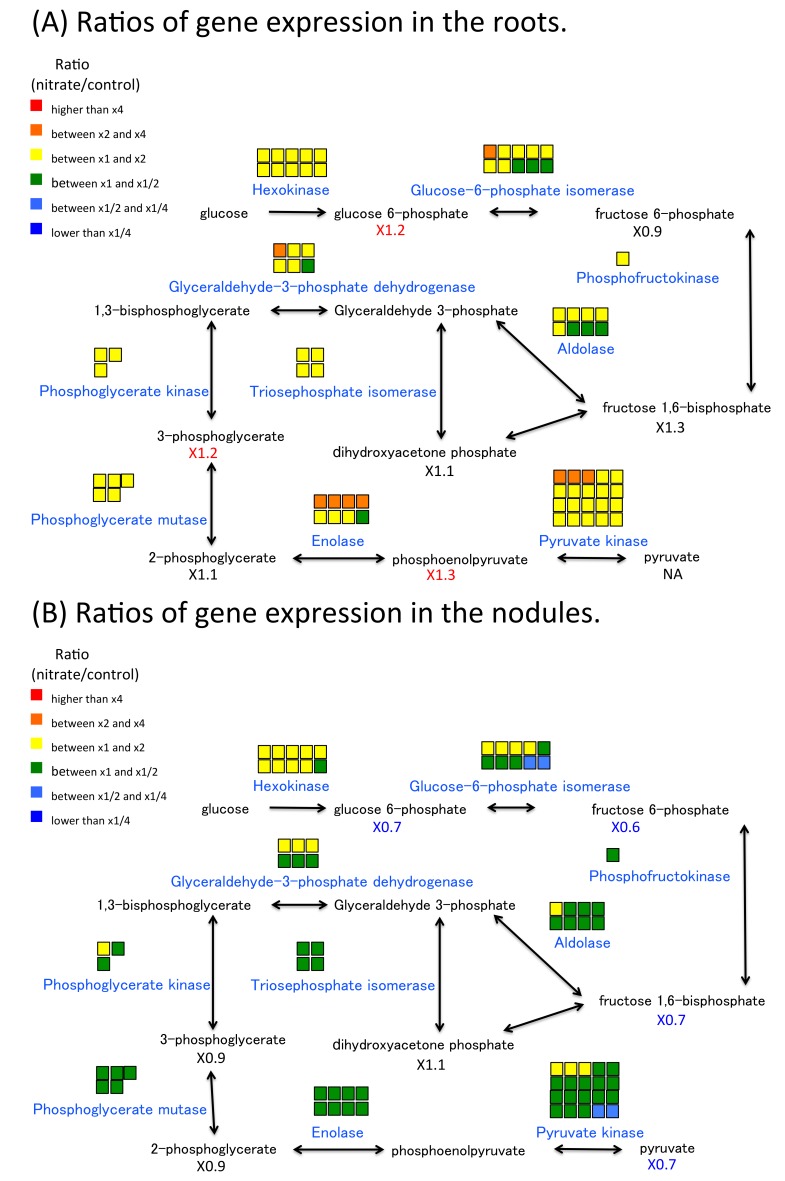
Ratios of gene expression and metabolite concentrations related to glycolysis in roots and root nodules after a 24-h 5-mM nitrate treatment. (**A**) Ratios of gene expression and metabolite concentrations in the roots. (**B**) Ratios of gene expression and metabolite concentrations in the nodules. The model shows the metabolic map of glycolysis in the root or nodule cells. The color of squares above enzyme name and the number under the compound abbreviation indicate the same as Figure 3.

**Figure 6:**
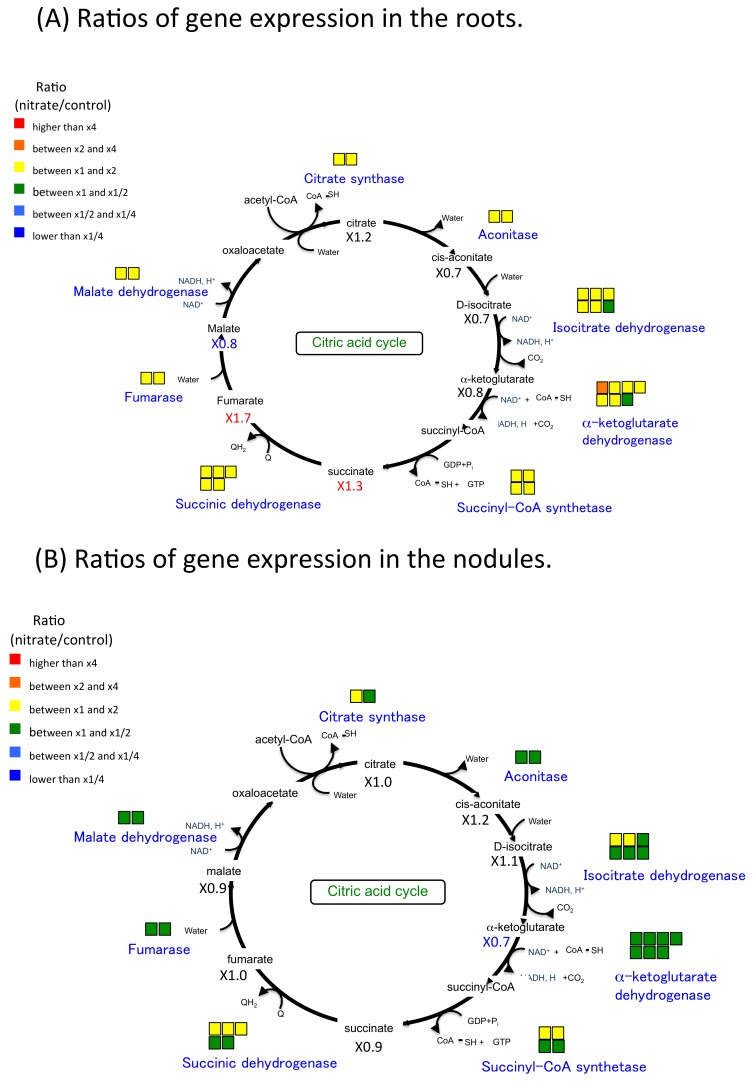
Ratios of gene expression and metabolite concentrations related to tricarboxylic acid (TCA) cycle in soybean roots and nodules after 24 h of the 5-mM nitrate treatment. (**A**) Ratios of gene expression and metabolite concentrations in the roots. (**B**) Ratios of gene expression and metabolite concentration in the nodules. The model shows the metabolic map of glycolysis in the root or nodule cells. The color of squares above enzyme name and the number under the compound abbreviation indicate the same as Figure 3.

**Figure 7:**
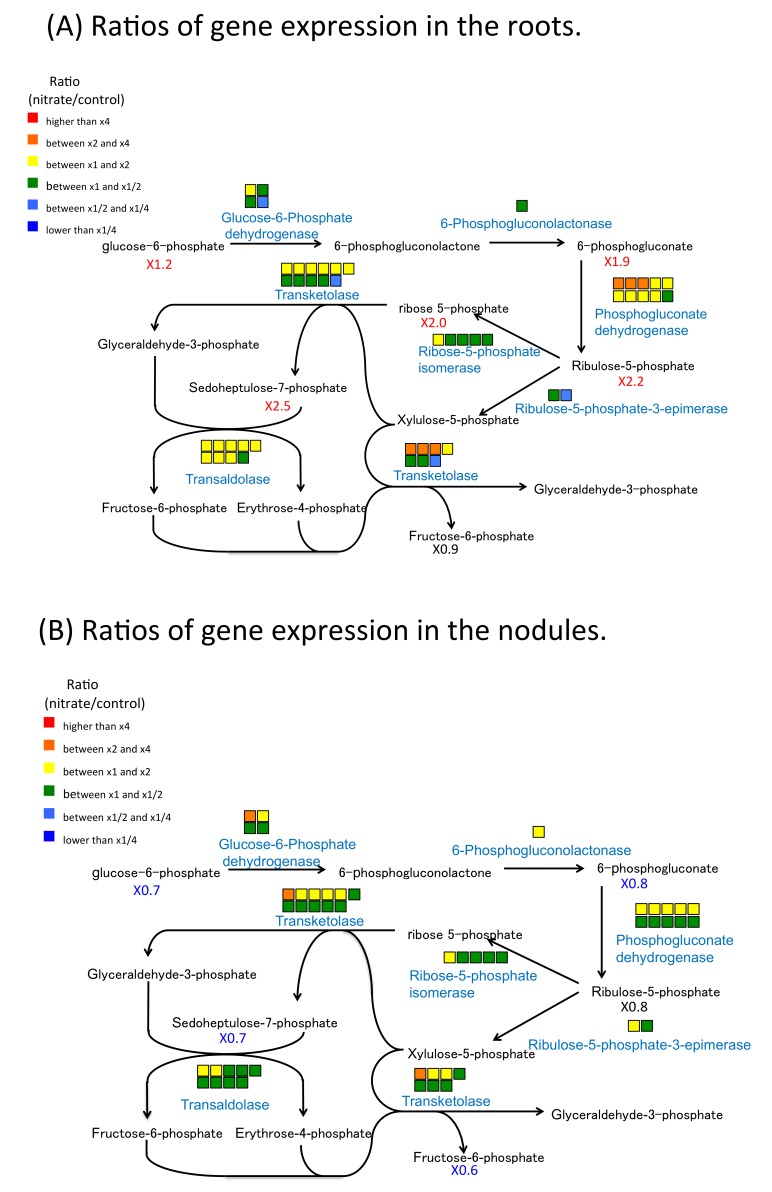
Ratios of gene expression and metabolite concentration related to the pentose phosphate pathway in soybean roots or nodules after 24 h of the 5-mM nitrate treatment. (**A**) Ratios of gene expression and metabolite concentration in the roots. (**B**) Ratios of gene expression and metabolite concentration in the nodules. The model shows the metabolic map of glycolysis in the root or nodule cells. The color of squares above enzyme name and the number under the compound abbreviation indicate the same as Figure 3.

Gene expression ratios in the roots and nodules as related to carbon metabolism are shown in Figure 5, Figure 6 and Figure 7. Most of the ratios (5 mM nitrate/control) for glycolysis (Figure 5A), the TCA cycle (Figure 6A), and pentose phosphate pathway (PPP) (Figure 7A) in the roots were c. x2. These results indicated that 5-mM nitrate treatment stimulated carbon metabolism in the roots. However, these ratios were not enhanced in the nodules, with many being less than x1 for glycolysis (Figure 5B), the TCA cycle (Figure 6B), and PPP (Figure 7B). These results indicated that nodule carbon metabolism was tended to decrease with the addition of 5 mM of nitrate for 24 h, which obviously differed from the metabolism trend observed for the roots. The compounds ratios of carbon compounds also tended to decreased in the nitrate-treated nodules.

## 3. Discussion

### 3.1. The Effect of Nitrate on Nitrate Absorption and Assimilation and Carbon Metabolism

Transcriptome and metabolome analyses of soybean roots and nodules treated with 5 mM of nitrate (NO_3_^−^) for 24 h revealed that supplying nitrate to the culture solution remarkably affected the expression of genes for enzymes involved in nitrate absorption and nitrogen assimilation in the roots. The similar trends were shown in the nodules for nitrate absorption and assimilation and asparagine synthase. Mizukoshi et al. [9] reported that the pathway of nitrate into nodule may be from nodule surface and not by transportation through xylem or phloem from the roots. Nitrate absorbed from the nodule surface might induce the nitrate transporters, nitrate reductase and nitrite reductase in nodules. Cabeza et al. [25] also reported that nitrate treatment on *Medicago trancatula* upregulated the genes of nitrate transporters, nitrate reductase and nitrite reductase. The expression of genes upregulated more than fourfold in the roots and nodules by the nitrate treatment were mainly classified to metabolism, especially with respect to amino acid transport and metabolism. The nitrate treatment also upregulated cellular processes, such as signal transduction mechanisms in the roots, whereas it did do the same in the nodules. By contrast, in the nodules, genes that were markedly downregulated by more than fourfold by the nitrate treatment were classified as energy production and conversion, and carbohydrate transport and metabolism. Although some nitrogen-assimilating enzymes such as AS were exceptionally enhanced in the nodules, in response to nitrate, the expression of other enzymes related to nitrogen metabolism was not markedly upregulated compared with roots. Nonetheless, the view that energy metabolism in the nodules may have been repressed following the application of 5 mM of nitrate is consistent with the lower concentrations of phosphorous compounds, namely ATP and NAD.

These results lend support to our posited hypothesis that the supply of nitrate to nodulated roots increased carbon consumption by promoting nitrogen absorption and assimilation in the roots, as well as promoting root growth; consequently, photoassimilate partitioning to the nodules was decreased [10,21]. When the nitrate was supplied to the nodulated roots of soybean plants, nodule growth stopped after just 1 day. This effect was reversible, however, because nodule growth quickly returned to its normal growth rate after withdrawing the nitrate from the culture solution [21]. In addition, the inhibitory effects of nitrate on nodule growth were partially alleviated by the addition of a 3% sucrose to the culture solution. Therefore, we proposed that the rapid and reversible inhibition of nodule growth and nitrogen fixation activity reflects changes in carbon partitioning from the nodules to the roots.

Gordon et al. [37] reported on the role of sucrose synthase (SS) in the nitrogen fixation of pea plants, by using a mutant line in which SS activity was markedly reduced. They found that SS, the enzyme to degrade sucrose primarily in the nodules transported through phloem, is essential for supplying C substrates for N_2_ fixation and for the development of functional pea nodules. The same research group demonstrated that soybean nodules exposed to nitrate exhibited weaker nitrogen fixation activity and the downregulated expression of SS transcripts just one day after a treatment with 10 mM of nitrate, whereas the SS activity and sucrose concentrations only decreased after 3 or 4 days of the 10-mM nitrate treatment [19]. Our results showed that the expression of SS genes was upregulated by nitrate supply in the roots but downregulated in the nodules, possibly reflecting crucial changes in the sucrose flux from nodules to roots.

### 3.2. The Effect of Nitrate on Asparagine and Ureide Metabolism

A distinct increase was observed in gene expression for asparagine synthetase (AS) in the nodules after the nitrate treatment (Figure 3B), with the concentration of asparagine in the nodules simultaneously increased 3.3-fold (Table 1). Asparagine accumulation in the nodules may have arisen directly by the promotion of asparagine synthesis; alternatively, it could be related to a reduction in the breakdown of asparagine by asparaginase or its decreased transport from the nodules to shoots. The expression of probesets of asparaginase was remarkably reduced in the nodules (Table S4) and roots (Table S3). It is interesting that the 5-mM nitrate treatment inhibits nitrogen fixation activity in Williams nodules [11], but asparagine accumulation was promoted in the nodules corresponding with upregulation of asparagine synthetase and downregulation of asparaginase. It has been reported that the major form of N compounds transported from soybean root nodules by nitrogen fixation are ureides, allantoin and allantoic acid, but those from roots absorbed nitrate is mainly asparagine [38]. The accumulation of asparagine in nodules may be related to the shifting from nitrogen fixation to nitrate assimilation.

On the other hand, the concentrations of ureides and precursors in nodules were changed by nitrate treatment for 24 h; xanthine (1.0-fold), uric acid (2.1-fold), allantoin (1.9-fold), allantoic acid (1.4-fold) (Table 1 and Table S15). The accumulation of uric acid, allantoin, and allanoic acid in the nodules treated with nitrate might be caused by decrease in ureide transport rather than promotion of ureide synthesis, because the genes of the enzymes related to ureide synthesis in nodules were not upregulated (Figure 4B). The concentrations of ureides and precursors in roots were also changed by nitrate treatment for 24 h; xanthine (N.A.), uric acid (N.A.), allantoin (1.0-fold), allantoic acid (3.7-fold) (Table 1 and Table S14). This may be related to the specific upregulation of uricase in the roots (Figure 4A), although the physiological meaning is unknown.

### 3.3. The Effect of Nitrate Treatment on Nodulin and Hormone Related Probesets

Leghemoglobin is the most abundant protein in soybean root nodules, and is an oxygen-binding protein that is essential for maintaining nitrogen fixation activity by keeping oxygen concentration low in infected region of nodules. Bojsen et al. [39] reported that there are six soybean leghemoglobin genes. Nishiwaki et al. [40] reported that the leghemoglobin concentration in soybean nodules was slightly decreased by nitrate supply. Recently, a protein analysis by 2-D PAGE showed that the leghemoglobin components remained stable in the soybean nodules treated with nitrate [22]. According to our array data, the six leghemoglobin probesets were not downregulated after 24 h of the supplied nitrate (Table S9), a result that contrasts with that for *Medicago truncatula* nodules [25]. This discrepancy may be due to plant species identity or the treatment methods used.

Early nodulin gene expressions of GmN70, GmN93, and GmN315 were significantly decreased in the nodules treated with nitrate (Table S10). Kouchi and Hata [41] reported that transcripts of GmN315 and GmN70 first appeared at 6–7 days just before nodule emergence, while GmN93 transcripts first appeared in the primary nodule meristem just below the root epidermis. These are early nodulins and thus different from late nodulins such as leghemoglobin and uricase II. These early nodulins are expressed during the infection and nodule formation processes, but the physiological meaning of repression in the mature nodules remain unknown.

## 4. Materials and Methods

### 4.1. Plant Growth and the Nitrate Treatment

Soybean seeds (*Glycine max* (L.) Merr., cultivar ‘Nourin No.2′), were surface-sterilized and inoculated with a suspension of *Bradyrhizobium diazoefficiens* (USDA 110) and sown in vermiculite. Five days after planting (DAP), 18 plants for transcriptome and 20 plants for metabolome analysis were transplanted into a nitrogen-free nutrient solution, as described before [22], in an 800-mL glass bottle covered with aluminum foil with continuous aeration. Plants were cultivated in a biophotochamber (MLR-350, Sanyo Electric Co. Ltd., Osaka, Japan) under 28 °C-day/18 °C-night temperatures, 55% relative humidity, and under a photon flux density of 228 mmol m^−2^ s^−1^ with a 16-h photoperiod and 8-h dark period. At 19 DAP, a 5-mM nitrate treatment was imposed for 24-h. The control plants were continually cultivated in an N-free culture solution. When this treatment ended, the lower half of the roots without visible nodules was used for the root analysis, to eliminate the mRNA or metabolites of the nodules, which were exclusively distributed in the upper part of the roots at this stage. Those nodules attached to the upper half of the root system with a diameter larger than 3 mm were collected and used for the nodule analysis. The fresh weights of the roots and nodules were separately determined and they were then immediately frozen in liquid nitrogen and stored at −80 °C.

### 4.2. Transcriptome Analysis

Soybean plants of the Japanese cultivar, Nourin No. 2, were used in this study because the array had already been constructed from this cultivar. Total RNA was extracted from the nodules and roots of the nitrate treated and control plants. The RNA was purified and then used in the cDNA microarrays that consisted of 22,674 nonredundant scaffolds. The quality of RNA was analyzed by Aglient 2100 Bionalalyzer. The Agilent Expression Array (a soybean oligoDNA microarray; Agilent Technologies, Ltd., Santa Clara, CA, USA) was used based on the sequencing and analysis of approximately 40,000 cDNA clones from a full-length-enriched cDNA library obtained from the soybean cultivar Nourin No.2 [34]. These microarrays were constructed in the format of a 44 K custom 60-mer oligonucleotide microarray from a data set of soybean full-length cDNAs and a public EST database [34]. The signal evaluation was done from the data calculated by Agilent Feature Extraction. About 80% of probes were detected to hybridize with transcripts. The expression signals were normalized for the average value as 2500 in each array. The DNA sequences of the probes are deposited in NCBI GEO database (design ID: 016772) (https://www.ncbi.nlm.nih.gov/geo/query/acc.cgi?acc=GPL15668). The sequences of all full-length cDNAs were deposited into the DDBJ (DNA Data Bank of Japan) with the accession numbers AK243693–AK246134 and AK285150–AK287419. The full-length cDNA information is also available at the URL of the RIKEN Center for Sustainable Resource Science: Integrated Genome Informatics Research Unit (http://spectra.psc.riken.jp/menta.cgi/rsoy/). Functional distribution of genes differentially expressed in soybean roots and nodules by the 5-mM nitrate treatment was analyzed using COGs database.

Soybean plants inoculated with the *Bradyrhizobium diazoefficiens* strain USDA110 were hydroponically cultivated under controlled conditions. At 19 DAP, the nine nodulated plants were treated with 5 mM nitrate for 24 h, after which they and the nine control plants were harvested and separated into roots and nodules, as described above. Roots and nodules from three plants were pooled with three biological replications. Total RNA was extracted with the RNA isoPlus (Fruit-mate for RNA purification, Takara Bio Inc. Kusatsu, Japan), and 500 ng of total RNA was used in the microarray analysis done by Takara Bio Inc. that used the microarray prepared from the cDNA of the soybean cultivar, Nourin No 2. The Cy3-labeled target cRNAs were prepared by using the Quick Amp Labeling Kit, one-color*3, and hybridized with the Agilent Expression Array, Agilent-016772 G. max (Soybean) Oligo Microarray: 4 arrays with 44 K), which contained about 42,000 probesets. Hybridization was done by using Gene Expression Hybridization Kit*4 (Agilent Technologies, Ltd., Santa Clara, CA, USA), and washing was then performed with the Gene Expression Wash Pack*5 (Agilent Technologies, Ltd., Santa Clara, CA, USA). Based on the signal evaluation by the Agilent Feature Extraction, about 81.0% of hybridization with the probes with cDNA from the control and 5-mM nitrate-treated roots with was detected with reliability, as were 83.5% and 82.2% of the hybridizations with cDNA from the control and 5-mM nitrate-treated nodules, respectively. Signals in each array were calibrated and the ratios of signals of nitrate treatment and control were calculated. Statistical analysis was done for calibrated signals of each probeset by the Student’s *t*-test. The obtained microarray data have been deposited in the Gene Expression Omnibus (GEO) database (https://www.ncbi.nlm.nih.gov.geo/) accession number GSE104582.

### 4.3. Metabolome Analysis

Ten soybean plants at 19 DAP were treated with 5 mM of nitrate for 24 h. The nitrate-treated and control plants were harvested, washed with de-ionized water, and separated into roots and nodules. Roots and nodules from two plants were pooled to form one replicate, and the analysis was performed with four replications for the roots and five replications for the nodules. Approximately 50 mg FW of the roots and nodules was quickly frozen with liquid nitrogen in the tube used for the extraction.

The frozen samples were send with dry ice and the metabolome analysis was outsourced to Human Metabolome Technologies, Inc. Tsuruoka, Japan. Samples were extracted with 500 µL of methanol with 50 µM of an internal standard (methionine sulfone, and 10-camphorsulfonic acid), and blended twice at 1500 rpm for 120 s using a tissue homogenizer (Micro Smash MS100R, Tomy Digital Biology Co., Ltd., Tokyo, Japan). Five-hundred µL of chloroform and 200 µL of pure water (Milli-Q water) were added to the sample homogenate, which was then separated by centrifugation at 2300 *g* at 4 °C for 5 min. Four hundred microliters of the water-soluble fraction were introduced into an ultrafiltration membrane tube (Millipore, Ultra free MC PLHCC HMT 5 kDa), and centrifuged at 9100 *g* at 4 °C for 120 min to remove proteins. The filtrate was evaporated to dryness and re-dissolved with 50 µL of Milli-Q water.

Samples were measured by either the cationic or anionic mode in the Agilent CE-TOFMS system equipped with an Agilent 6210 Time of Flight mass spectrometer, Agilent 1100 isocratic HPLC pump, Agilent G1603A CE-MS adapter kit, and Agilent G1607A CE-ESI-MS sprayer kit (Agilent Technologies, Waldronn, Germany). The systems were controlled by Agilent G2201AA ChemStation software version B.0.01 for CE (Agilent Technologies, Waldronn, Germany). The metabolites were analyzed by using a fused silica capillary (50 µm *i.d.* 80 cm total length). The cation analysis was performed by the following conditions: Run buffer, Cation Buffer Solution (Solution ID: H3301-1001), Rinse buffer: Cation Buffer Solution (Solution ID: H3301-1001), Sample injection: Pressure injection 50 mbar, 10 s. CE voltage: Positive, 27 kV, MS ionization: ESI Positive, MS capillary voltage: 4000 V, MS scan range: *m*/*z* 50–1000, Sheath liquid: HMT Sheath Liquid (Solution ID: H3301-1020). The anion analysis was performed by the following conditions: Run buffer, Anion Buffer Solution (Solution ID: I3302-1023), Rinse buffer: Anion Buffer Solution (Solution ID: I3302-1023), Sample injection: Pressure injection 50 mbar, 25 s (approximately 10 nL), CE voltage: Positive, 30 kV, MS ionization: ESI Negative, MS capillary voltage: 3500 V, MS scan range: *m*/*z* 50–1000, Sheath liquid: HMT Sheath Liquid (Solution ID: H3301-1020). Other conditions were as same as described previously [42].

The peaks of CE-TOFMS were detected by integration software MasterHands ver.2.13.0.8h (Keio University, Tsuruoka, Japan) in order to obtain peak information including *m*/*z*, migration time for CE-TOFMS measurement (MT) and peak area [43]. The tolerance range for the peak annotation was configured at ±0.5 min for MT and ±10 ppm for *m/z*. The relative peak area was calculated from the peak area of compounds divided by the peak area of the internal standards. The concentration ratio (nitrate/control) of each compound was calculated. Statistical analysis was done for the concentrations of each compound by using the Welch’s *t*-test.

Transcriptome and metabolome analysis in the present research revealed that nitrate strongly promotes nitrogen and carbon metabolism in the roots. Nitrate tends to repress the carbon and nitrogen metabolism in the nodules, although it enhanced nitrate transport and assimilation and asparagine synthesis by the promotion of asparagine synthase and repression of asparaginase. The biological meanings of the promotion of asparagine synthase and asparagine accumulation in nodules are not understood. This may be induced by the nitrate assimilation in the nodules. In addition, the effects of nitrate on leghemoglobin and phytohormone-related genes are not remarkable both in the nodules and roots after 24 h of nitrate supply.

## Figures and Tables

**Table 1:** Comparison of the ratios of concentrations of nitrogen compounds in soybean roots and nodules treated with 5 mM of nitrate for 24 h and the control plants.

Nitrogen Compound	Roots	Nodules
Allantoic acid	3.1 ***	1.4 *
Glutamine	2.4 *	1.6 *
Asparagine	2.1 *	3.3
Alanine	2.0 ***	0.9
Homoserine	2.8 *	0.8
2-Aminobutyric acid	1.9 **	0.8 *
Glutamic acid	1.8 **	1.1
Aspartic acid	1.8 *	1.4 *
Allantoin	1.0	1.9 **
*N*-Acetylglucosamine	1.1	3.6 *

Notes: The symbol (*, **, ***) after each number indicates the statistical significance of the metabolite concentration between the nitrate treatment and control at *p* < 0.05, 0.01, 0.001 levels by Welch’s *t*-test. Red color stands for Ratio (Nitrate/Control) > 3; Orange stands for 2 ≤ Ratio (Nitrate/Control) ≤ 3; Yellow stands for 1 ≤ Ratio (Nitrate/Control) < 2; Blue stands for Ratio (Nitrate/Control) < 1.

**Table 2:** Comparison of the ratios of concentrations of phosphorous compounds in soybean roots and nodules treated with 5 mM of nitrate for 24 h and the control plants.

Phosphorous Compound	Roots	Nodules
ATP	3.9 **	0.7
ADP	2.9 *	0.9
GMP	2.8 **	N.A.
CDP	2.4	0.8
UDP	2.1 *	0.9
CTP	2.1 *	N.A.
Sedoheptulose 7-phosphate	2.5 **	0.7 *
Ribulose 5-phosphate	2.2 ***	0.8
NAD^+^	2.2 *	0.9
Ribose 5-phosphate	2.0 ***	0.8
6-Phosphogluconic acid	1.9 ***	0.8 **
Spermidine	1.8 *	1.2
*O*-Acetylserine	1.7 *	N.A.
AMP	1.6 *	1.3
UMP	1.6 *	0.8
CMP	1.5 *	1.0
UDP-glucuronic acid	1.4 *	0.7
Phosphoenolpyruvic acid	1.3 *	0.9
Glucose 1-phosphate	1.2 *	0.8 *
Glucose 6-phosphate	1.2 *	1.0
3-Phosphoglyceric acid	1.2 *	0.9

Notes: The symbol (*, **, ***) after each number indicates the statistical significance of the metabolite concentration between the nitrate treatment and control at *p* < 0.05, 0.01, 0.001 levels by Welch’s *t*-test. N.A.: data is not available. Red color stands for Ratio (Nitrate/Control) > 3; Orange stands for 2 ≤ Ratio (Nitrate/Control) ≤ 3; Yellow stands for 1 ≤ Ratio (Nitrate/Control) < 2; Blue stands for Ratio (Nitrate/Control) < 1.

**Table 3:** Comparison of the ratios of concentrations of organic acids in soybean roots and nodules treated with 5 mM of nitrate for 24 h and the control plants.

Organic Acid	Roots	Nodules
2-Isopropylmalic acid	2.4 *	1.0
2-Aminoadipic acid	2.1 **	1.1
*N*-Acetylglutamic acid	1.9 **	1.1
Citramalic acid	1.9 **	0.9
Fumaric acid	1.7 ***	1.0
Argininosuccinic acid	1.4 **	1.3 *
Succinic acid	1.3 *	0.9
Phosphoenolpyruvic acid	1.3 *	0.9
Malic acid	0.8 *	0.9

Notes: The symbol (*, **, ***) after each number indicates the statistical significance of the metabolite concentration between the nitrate treatment and control at *p* < 0.05, 0.01, 0.001 levels by Welch’s *t*-test. Red color stands for Ratio (Nitrate/Control) > 3; Orange stands for 2 ≤ Ratio (Nitrate/Control) ≤ 3; Yellow stands for 1 ≤ Ratio (Nitrate/Control) < 2; Blue stands for Ratio (Nitrate/Control) < 1.

## Supplementary Materials

The following are available online at http://www.mdpi.com/2223-7747/7/2/32/s1, Figure S1. Ratios of auxin-related gene expression in soybean roots and nodules by the nitrate treatment. Table S1. List of the upregulated probesets with ratios of nitrate/control more than two-fold in the roots after 24 h of the 5-mM nitrate treatment. Table S2. List of the upregulated probesets with ratios of nitrate/control more than two-fold in the nodules after 24 h of the 5-mM nitrate treatment. Table S3. List of the downregulated probesets with ratios of control/nitrate more than two-fold in the roots after 24 h of the 5-mM nitrate treatment. Table S4. List of the downregulated probesets with the ratios of control/nitrate more than two-fold in the nodules after 24 h of the 5-mM nitrate treatment. Table S5. List of root-specific genes enhanced by the nitrate treatment. Table S6. List of root-specific genes decreased by the nitrate treatment. Table S7. List of nodule-specific probesets enhanced by the nitrate treatment. Table S8. List of nodule-specific probesets downregulated by the nitrate treatment. Table S9. Effect on gene expression of leghemoglobin by the nitrate treatment in soybean roots and nodules. Table S10. Effect on gene expression of the nodulin genes by the nitrate treatment in soybean roots and nodules. Table S11. List of the name, KEGG ID, HMDB ID, *m*/*z*, MT and relative area of each compound in the roots and nodules in control and nitrate treatments. Table S12. Concentration of soluble compounds in roots and nodules treated with nitrate. Table S13. List of ratios of the concentration of each compound in the nodules vs. roots in control treatment. Table S14. List of ratios of the concentration of each compound in the roots in 5-mM nitrate treatment vs. control. Table S15. List of ratios of the concentration of each compound in nodules in 5-mM nitrate treatment vs. control.

## Abbreviations

AICAR	5-aminoimidazole-4-carboxamide ribonucleotide
AIR	5-aminoimidazole ribotide
AON	autoregulation of nodulation
AS	asparagine synthetase
Asn	asparagine
Asp	aspartate
CAIR	5′-phosphoribosyl-4-carboxy-5-aminoimidazole
FAICAR	5-formamidoimidazole-4-carboxamide ribotide
FGAM	5′-phosphoribosylformylglycinamideine
FGAR	phosphoribosyl-*N*-formylglycineamide
Frc	fructose
GAR	glycineamide ribonucleotide
G-6-P DH	glucose-6-phosphate dehydrogenase
Gln	glutamine
Glu	glutamate
GMP	guanosine 5′-monophosphate
GOGAT	glutamate synthase
GS	glutamine synthetase
IMP	inosinic acid
NAR2	high-affinity nitrate transporter-activating protein
(d)NDP	(deoxy) nucleotidediphosphate
NR	nitrate reductase
NRT	nitrate transporter
(d)NTP	(deoxy) nucleotidetriphosphate
OAA	oxaloacetate
2-OG	2-oxoglutarate
PEP	phosphoenolpyruvate
PEPC	phosphoenol pyruvate carboxylase
PPP	pentose phosphate pathway
R-5-P	ribose-5-phosphate
PRPP	5-phosphoribosyl 1-diphosphate
PRA	5-phosphoribosylamine
Pyr	pyruvate
SAICAR	phosphoribosylaminoimidazolesuccinocarboxyamide
SS	sucrose synthase
Suc	sucrose
